# Licochalcone A Induces Uterine Leiomyoma Cell Apoptosis via the ROS-Mediated JNK Activation of the GRP78/NRF2 Pathway In Vitro and In Vivo

**DOI:** 10.3390/antiox14020148

**Published:** 2025-01-27

**Authors:** Hung-Ju Chien, Huang-Ming Hu, Su-Ju Tsai, Chu-Liang Lin, Shun-Fa Yang, Ju-Kai Chen, Chung-Jung Liu, Yi-Hsien Hsieh

**Affiliations:** 1 Department of Obstetrics and Gynecology, Changhua Christian Hospital, Changhua 50006, Taiwan; 180973@cch.org.tw; 2 Division of Gastroenterology, Department of Internal Medicine, Kaohsiung Medical University Hospital, Kaohsiung Medical University, Kaohsiung 807378, Taiwan; 990361@kmuh.org.tw; 3 Department of Internal Medicine, Faculty of Medicine, College of Medicine, Kaohsiung Medical University, Kaohsiung 807378, Taiwan; 4 Department of Internal Medicine, Kaohsiung Municipal Ta-Tung Hospital, Kaohsiung 807378, Taiwan; 5 Department of Physical Medicine and Rehabilitation, Chung Shan Medical University School of Medicine, Taichung City 40201, Taiwan; sujutsai@csmu.edu.tw; 6 Department of Physical Medicine and Rehabilitation, Chung Shan Medical University Hospital, Taichung City 40201, Taiwan; 7 Institute of Medicine, Chung Shan Medical University, Taichung City 40201, Taiwan; like751019@live.csmu.edu.tw (C.-L.L.); ysf@csmu.edu.tw (S.-F.Y.); hospital.tc@blood.org.tw (J.-K.C.); 8 Department of Medical Research, Chung Shan Medical University Hospital, Taichung City 40201, Taiwan; 9 Regenerative Medicine and Cell Therapy Research Center, Kaohsiung Medical University, Kaohsiung 807378, Taiwan

**Keywords:** licochalcone A, uterine leiomyomas, ROS, ER stress, apoptosis, JNK, NRF2, GRP78

## Abstract

Licochalcone A (LicoA) possesses anti-tumor properties. However, the potential therapeutic effect of LicoA on uterine leiomyomas (ULs) remains unknown. In this study, the effects of LicoA on the proliferation of ULs and its underlying mechanism were explored. LicoA treatment significantly decreased the viability of uterine smooth muscle cells (UtSMCs) and ELT3 cells in a dose-dependent manner. The induction of ELT3 cell apoptosis by LicoA was accompanied by the increased generation of reactive oxygen species (ROS), elevated endoplasmic reticulum (ER) stress (GRP78/IRE1α/ATF6/CHOP), and the increased expression of proapoptotic proteins (c-caspase-3, c-caspase-9, and c-PARP). The ability of Z-VAD-FMK (a caspase inhibitor) and n-acetylcysteine (NAC; a cell membrane permeable antioxidant) to reverse LicoA-induced ROS-mediated ER stress pathways also observed. Furthermore, GRP78 or JNK knockdown was involved in LicoA-induced ROS-mediated ER stress and apoptosis in ELT3 cells. In immunodeficient mice, LicoA significantly suppressed the growth of ELT3 tumor cells, without toxicity. This study is the first to show that LicoA exerts anti-leiomyoma effects via the modulation of ROS-mediated ER stress-induced apoptosis through the JNK/GRP78/NRF2 signaling pathway.

## 1. Introduction

Uterine leiomyoma (UL), a common noncancerous tumor, comprises muscle and tissue that form in or on the uterine wall. UL is the most common female reproductive disorder, occurring in approximately 80% of women of childbearing age [1,2]. The clinical complications of UL include menorrhagia, severe pelvic pain, frequent urination, and infertility [3,4,5]. Myomectomy and hysterectomy have traditionally been the surgical approaches used to treat UL. However, the cumulative rate of UL recurrence within 5 years of surgery is 62% [6]. Genetic and non-genetic factors have been reported to contribute to the development of UL, including genetic polymorphisms, menopausal status, diet, age, alcohol and tobacco use, and body mass index [7,8,9]. Uterine fibroids can contribute to the development of new targeted therapies. At present, few therapies based on non-invasive agents are available for the treatment of UL.

Basal levels of reactive oxygen species (ROS) are critical for the physiological functioning of cells. ROS such as superoxide and hydrogen peroxide participate in autophagy, differentiation, and immune responses [10]. However, the excessive production of intracellular ROS can reduce the permeability of the mitochondrial membrane and the stability of genomic DNA, proteins, and lipids, ultimately leading to tumorigenesis [11,12]. Superoxide, hydrogen peroxide, and hydroxyl radicals have been studied extensively in relation to a variety of cancers [13]. The transcription factor NRF2 is a primary sensor of oxidative stress and a regulator of redox homeostasis. NRF2 activity is regulated by multiple extracellular and intracellular signals [14], including phosphorylation, via signal transduction pathways such as PI3K/Akt, PKC, ERK, and JNK [15]. NRF2 induces the expression of heme oxygenase-1 (HO-1) under oxidative stress conditions [16]. ROS are known to induce apoptosis via endoplasmic reticulum (ER) stress [17]. The ER is responsible for the synthesis, correct folding, and modification of proteins. An increase in ER stress caused by external factors or environmental damage can disrupt the correct folding of proteins [18,19]. The excessive formation of ROS has been shown to cause apoptosis and induce cell cycle arrest in many types of cancer cells [20,21] via the ER stress-mediated unfolded protein response (UPR) [22,23]. These findings suggest that natural compounds that induce excess ROS generation and ER stress could be employed to treat UL.

Medicines based on the natural components of plants have been investigated as therapeutic strategies for the treatment of tumors [24]. LicoA, a naturally occurring chalcone extracted from licorice, exerts diverse pharmacological effects [25] against inflammation [26], angiogenesis [27], and tumor progression [28,29]. The use of herbal products to improve UL is considered an alternative treatment. Several herbal products have been reported to reduce the risk of UL [30,31,32]. In this study, we investigated the ability of LicoA to treat UL using ELT3 cells to identify the therapeutic agents and mechanisms potentially associated with UL.

## 2. Materials and Methods

### 2.1. Reagents

Licochalcone A (LicoA, BP0855) was purchased from Chengdu Biopurify Phytochemicals Ltd. (Chengdu, China) and prepared as a stock concentration of 50 mM. The antibodies used for western blot analysis are as follows: GRP78 (#3177; 1:1000), IRE1α (#3294, 1:1000), c-PARP (#9542, 1:1000). These were purchased from Cell Signaling Technology (Danvers, MA, USA). c-caspase-3 (sc-56053, 1:1000), c-caspase-9 (sc-133109, 1:1000), p-JNK (sc-81502, 1:1000), JNK (sc-7345, 1:1000), p-p38 MAPK (sc-101759, 1:1000), p38 MAPK (sc-7972, 1:1000), p-ERK (sc-101761, 1:1000), ERK (sc-514302, 1:1000), ATF6 (sc-166659, 1:1000) and β-actin (sc-517582, 1:5000) were purchased from Santa Cruz Biotechnology (Dallas, Texas, USA). CHOP (A20987, 1:1000) was purchased from ABclonal Technology (Woburn, MA, USA), and phospho-NRF2 (PA5-67520, 1:1000) and NRF2 (MA5-38583) were purchased from Thermo Fisher Sci-entific (Waltham, MA, USA). Ki-67 (ab15580, 1:100) was purchased from Abcam (Waltham, MA, USA). The DCFDA Cellular ROS Detection kit (ab113851), caspase-3 activity assay (ab39401), caspase-9 activity assay (ab65608), and LDH activity assay (ab65393) were purchased from Abcam Company (Cambridge, UK). The ER-ID assay (ENZ-51025) was purchased from Enzo Life Sciences, Inc. (Farmingdale, NY, USA). The RNAiMAX transfection reagent and Annexin V/PI assay kit were purchased from Thermo Fisher Scientific (Waltham, MA, USA).

### 2.2. Cell Culture

Eker leiomyoma tumor-3 (ELT3) cells were used in this study due to their distinct genetic and biological characteristics, including a germline defect in the TSC2 tumor suppressor gene, a high proliferative rate, and their potential application in in vivo tumorigenesis uterine leiomyoma mice models. In addition, UtSMCs are normal smooth muscle cells and are often used as a control model to assess the cytotoxic effects of various drug treatments. Uterine smooth muscle cells (UtSMCs) and ELT3 leiomyoma cells were gifts from Dr. Shih-Min Hsia (Taipei Medical University) and have been previously described in a published study [33]. Both cell lines were maintained in DMEM/F12 media (Cytiva; Marlborough, MA, USA) with 10% fetal bovine serum (FBS, Cytiva, Marlborough, MA, USA) and P/S mix reagent (100 U/mL, Thermo Fisher Scientific, Waltham, MA, USA). The cells were cultured in a humidified atmosphere containing 5% CO_2_ at 37 °C.

### 2.3. siRNA Transfection

Cells were transfected with JNK siRNA (10 nM of si-JNK) or CHOP siRNA (10 nM of si-CHOP) using the RNAiMAX transfection reagent (Thermo Fisher Scientific, Waltham, MA, USA) for 24 h; this was followed by the addition of LicoA and incubation for 24 h. Total proteins were extracted to determine the effect of LicoA on cells with and without gene knockdown.

### 2.4. Cell Viability Assay

The cell viability was assessed using the MTT assay, as described previously [34]. The absorbance of blue crystals in cells treated with different concentrations of LicoA (0–60 μM) was determined using an ELISA reader machine (wavelength, 570 nm). The rate of cell growth was calculated as follows: (LicoA-treated cells/untreated cells) × 100%.

### 2.5. Immunoblot Assays

Cells were treated with LicoA for 24 h and subsequently lysed using a protein lysis buffer containing a cocktail (proteinase inhibitor, 100×) and 1% NP40. The lysates were incubated on ice for 20 min and the total proteins were quantified. The total proteins were then separated on a 10% SDS-PAGE gel for 60 min and blocked with 2% BSA in TBST buffer for 1 h. Membranes were incubated with the indicated antibodies at 4 °C overnight. After washing, the membranes were incubated with HRP-IgG antibodies for 1 h. Proteins were detected using a chemiluminescence detection reagent, visualized, and quantified using the ImageQuant LAS 4000 Mini imaging system. 

### 2.6. ROS Detection Assay

After treatment with various concentrations of LicoA, the cells were fluorescence stained using the DCFH-DA ROS assay. Fluorescence was analyzed using the ImageXpress Pico automated cell imaging system (Molecular Devices, LLC, San Jose, CA, USA). The increase in fluorescence intensity over baseline directly corresponded to the number of intracellular ROS produced.

### 2.7. LDH Cytotoxicity Assay

Lactate dehydrogenase (LDH) release was used to measure the cell death rate. Cells were treated with various concentrations of LicoA for 24 h, and the amount of LDH released was determined using the LDH Assay Kit (Cytotoxicity) following the manufacturer’s instructions.

### 2.8. ER Stress Detection Assay

After treating the cells with various concentrations of LicoA, the ER-ID Green Detection Kit (ENZ-51025) was used to selectively fluorescently stain the endoplasmic reticulum (ER) according to the manufacturer’s instructions [34]. Fluorescence was then detected using the ImageXpress Pico automated cell imaging system for ER stress analysis, with increases in the fluorescence intensity directly corresponding to the amount of ER stress.

### 2.9. Annexin V/PI Staining for Apoptosis Detection

After being treated with various concentrations of LicoA, the cells were detached using trypsin–EDTA and centrifuged at 1000 rpm for 5 min at room temperature. The cells were then stained using the Annexin V and Dead Cell Kit and analyzed for apoptosis via flow cytometry, using the Muse Cell Analyzer (Cytek® Biosciences, Fremont, CA, USA).

### 2.10. Caspase 3 and 9 Activity Assays

Lysates of cells treated with LicoA were analyzed for caspase-3 and caspase-9 activity using the caspase-3 and caspase-9 assay kit, respectively, following the manufacturer’s instructions.

### 2.11. Animal Experiments and Immunohistochemistry Staining

Animal experiments were conducted in accordance with the Guidelines for the Care and Use of Laboratory Animals of the Council of Agriculture, Executive Yuan, Taiwan, and were approved by the Institutional Animal Care and Use Committee (IACUC Approval No. 112003) of Chung Shan Medical University, Taiwan. The experimental animals were five-week-old immunodeficient BALB/c nude mice, obtained from the National Laboratory Animal Center, Taipei, Taiwan. The mice were randomly divided into three groups: (1) control group (*n* = 5), (2) LicoA 10 mg/kg (*n* = 5), and (3) LicoA 20 mg/kg (*n* = 5). The treatments were administered via gavage twice a week for one month, with the tumor growth measured once a week. Organ tissues (liver, spleen, kidney, and heart) were fixed in 10% formalin, embedded in paraffin, and sectioned. Hematoxylin and eosin (H&E) staining was performed in order to analyze tissue morphology and evaluate the safety of the drug. The proliferation of the LicoA-treated cells was determined using Ki-67 antibodies (ab15580, 1:100) for immunohistochemistry (IHC) staining, which was observed under an optical microscope.

### 2.12. Statistical Analysis

Statistical analyses were performed using SPSS 20 statistical software, as described in a previous study [35]. The significance of differences between the groups was determined using Student’s *t* test, with the statistical significance defined as *p* < 0.05 or *p* < 0.01.

## 3. Results

### 3.1. Effect of LicoA on the Growth of UtSMCs and ELT3 Cells

**Figure 1:**
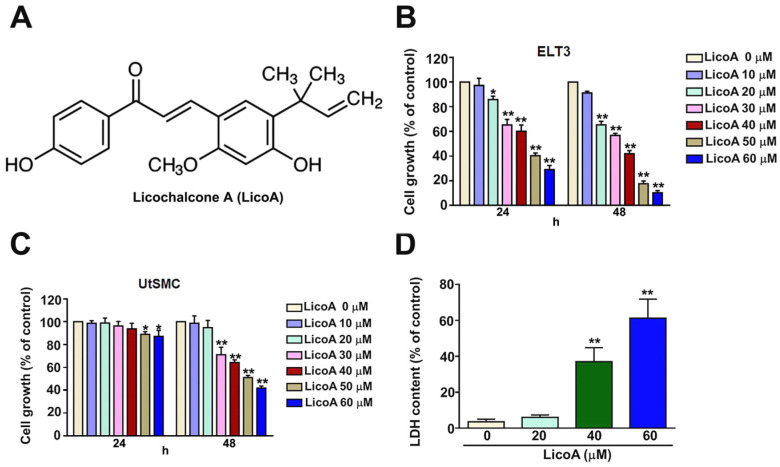
Effects of licochalcone A (LicoA) on the viability of uterine smooth muscle cells (UtSMCs) and uterine leiomyoma ELT3 cells. (**A**) Structure of licochalcone A (LicoA). (**B**,**C**) The MTT assay was performed to measure the viability of UtSMCs (2 × 10^4^ cells per well) and ELT3 cells (2 × 10^4^ cells per well) seeded in 24-well plates. Both cell types were exposed to LicoA at a range of doses (0, 10, 20, 30, 40, 50, or 60 μM) for 24 and 48 h. (**D**) The cytotoxicity of LicoA in ELT3 cells, as determined by the cytotoxicity LDH assay. * *p* < 0.05; ** *p* < 0.01 versus control (means ± SE; *n* = 3).

The structure of LicoA is shown in Figure 1A. ELT3 cells and UtSMCs were exposed to various concentrations of LicoA (0, 10, 20, 30, 40, 50, and 60 µM) for 24 and 48 h. The MTT assay results showed that treatment with LicoA at concentrations above 20 µM for 24 and 48 h significantly reduced the growth of ELT3 cells (Figure 1B). After 24 h, 50 and 60 µM of LicoA slightly reduced the growth of UtSMCs. However, LicoA concentrations above 30 µM for 48 h inhibited the growth of UtSMCs (Figure 1C). The LDH cytotoxicity assay revealed that LicoA was toxic to ELT3 cells in a dose-dependent manner (Figure 1D). These results suggest that LicoA exhibits highly selective and specific anti-tumor activity against ELT3 cells.

### 3.2. LicoA Induces Apoptosis of ELT3 Cells

**Figure 2:**
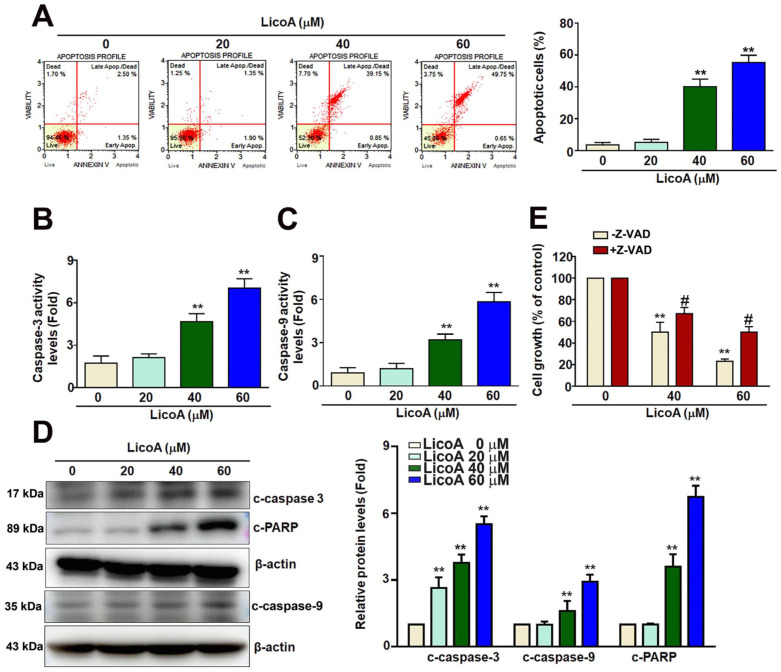
Effect of LicoA on apoptosis in ELT3 cells. (**A**) Apoptosis profile of ELT3 cells exposed to various concentrations of LicoA (0, 20, 40, and 60 μM) for 24 h; cells were stained with Annexin V and propidium iodide. The number of annexin V-positive cells was determined via flow cytometry. (**B**,**C**) An increase in the activity of caspase-3 and caspase-9 was observed after the treatment of ELT3 cells with LicoA. (**D**) The active forms of c-caspase-3, c-caspase-9, and c-PARP that were induced by LicoA in ELT3 cells were measured via Western blotting. (**E**) Caspase inhibitor Z-VAD-FMK (Z-VAD) co-treatment with LicoA was performed to detect the cell growth of ELT3 cells via the MTT assay. ** *p* < 0.01 versus control; # *p* < 0.05 versus LicoA treatment.

Annexin V staining analysis showed that 40 and 60 μM of LicoA significantly increased the apoptosis of ELT3 cells in a dose-dependent manner from 5.0% to 65.7% (Figure 2A). The ELT3 cells treated with LicoA exhibited increased caspase-3 and caspase-9 activity (Figure 2B,C). Consistent with the caspase activity data, the cleaved forms of caspase-3, caspase-9, and the PARP protein were elevated in LicoA-treated ELT3 cells (Figure 2D). Thus, we examined whether caspase-3/-9 pathways mediated the LicoA-induced apoptosis of ELT3 cells. Co-treatment with Z-VAD-FMK (Z-VAD, a pan-caspase inhibitor) and LicoA (40 and 60 μM) partially reversed the growth of ELT3 cells (Figure 2E). These findings suggest that LicoA induces apoptosis via caspase-3/-9/-PARP pathways.

### 3.3. LicoA Induces ROS Generation Through the Activation of NRF2-Mediated Apoptosis in ELT3 Cells

**Figure 3:**
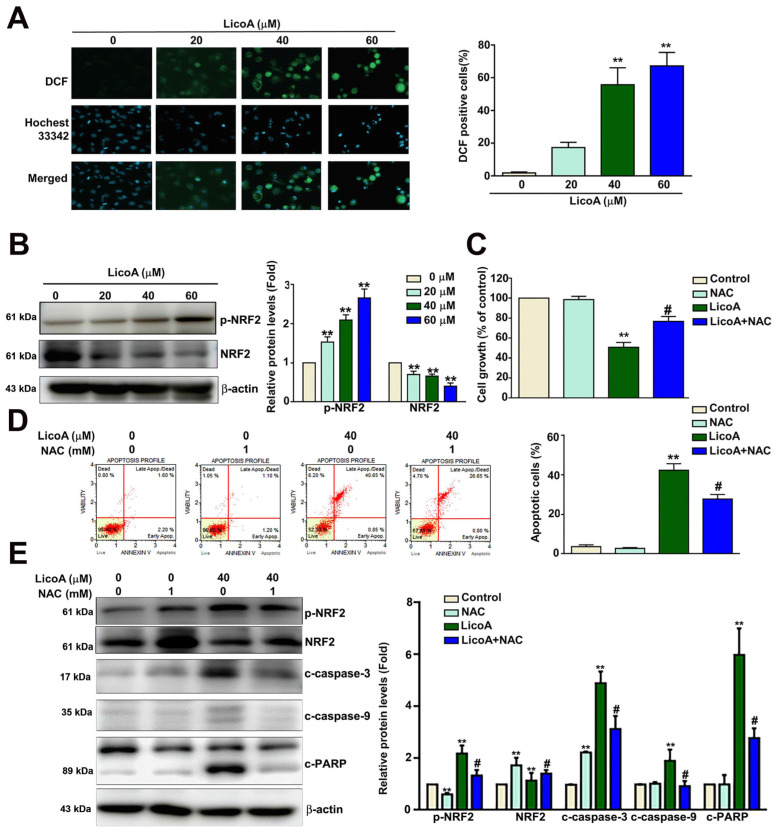
Effect of NAC on LicoA-induced ROS-responsive p-NRF2 and apoptosis in ELT3 cells. (**A**) The DCFDA (2′,7′-dichlorofluorescin diacetate)–ROS detection assay was performed to measure the generation of ROS in ELT3 cells exposed to various concentrations of LicoA (0, 20, 40, and 60 μM) for 24 h. Scale bar = 50 μm. (**B**) The expression of phosphorylated NRF2 (p-NRF2) and total NRF2 (NRF2) in ELT3 cells treated with LicoA, as determined via Western blotting. (**C**) The cell growth of ELT3 cells exposed to LicoA (40 μM) with or without NAC (1 mM), as determined via the MTT assay. (**D**) Annexin V/PI staining was used to assess the apoptotic effect of LicoA (40 μM) with or without NAC (1 mM) on ELT3 cells. (**E**) The expression of p-NRF2, NRF2, c-caspase-3, c-caspase-9, and c-PARP was measured via Western blotting. ** *p* < 0.01 versus control. # *p* < 0.05 versus LicoA treatment.

The intracellular ROS levels were determined using the DCF fluorescence content [36]. The intracellular DCF fluorescence of the ELT3 cells treated with LicoA (0, 20, 40, and 60 µM) for 24 h increased significantly, from 2.4% to 62.3% (Figure 3A). The treatment of ELT3 cells with LicoA significantly increased the level of phosphorylated NRF2 and decreased the expression of NRF2 (Figure 3B). The anti-proliferative effects of LicoA on ELT3 cells were significantly reversed in the presence of 1 mM of n-acetylcysteine (NAC, a cell membrane-permeable antioxidant) (Figure 3C) and significantly decreased the prevalence of LicoA-induced cell apoptosis, from 41.4% to 27.4% (Figure 3D). Western blot analysis showed that the LicoA-induced increases in phosphorylated NRF2 (p-NRF2), c-caspase-3, c-caspase-9, and c-PARP were dramatically inhibited by treatment with NAC (1 mM) (Figure 3E). These findings indicate that the anti-growth effect of LicoA on leiomyoma cells involves a ROS-mediated mechanism.

### 3.4. LicoA Induces ER Stress-Mediated GRP78/p-NRF2-Targeted Cell Apoptosis in ELT3 Cells

**Figure 4:**
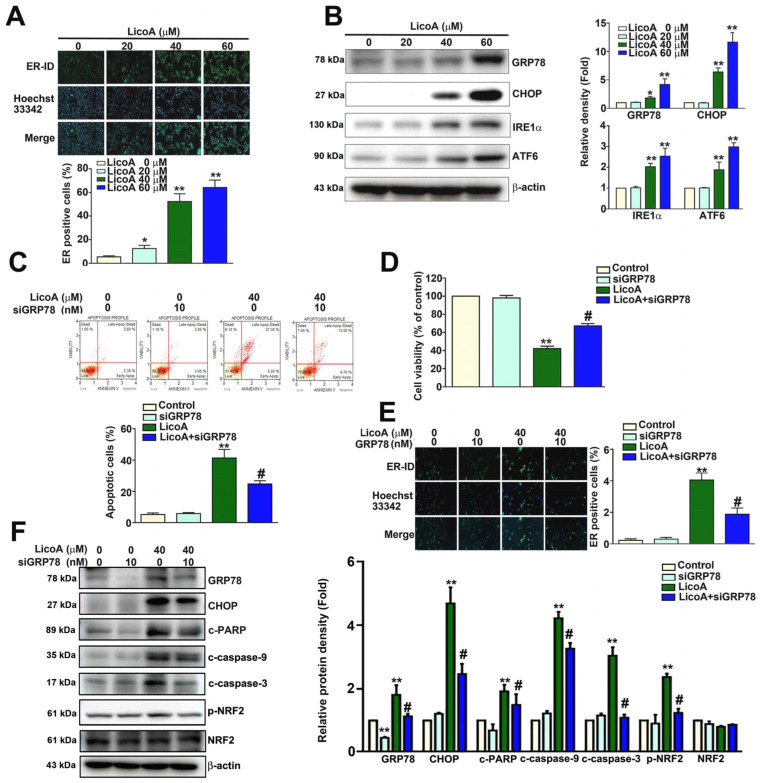
LicoA induction of ER stress-mediated cell apoptosis via the targeting of GRP78 in ELT3 cells. (**A**) ELT3 cells exposed to various concentrations of LicoA (0, 20, 40, and 60 μM) for 24 h were subjected to ER-ID immunofluorescence staining. The endoplasmic reticulum of live ELT3 cells was stained with ER-ID green dye and Hoechst 33342 nuclear stain (Blue); the images were merged to yield a composite image (Merge), scale bar = 50 μm. (**B**) LicoA-induced changes in ER stress-related proteins (GRP78/IRE1α/ATF6/CHOP) in ELT3 cells, as determined via Western blotting. (**C**) Annexin V/PI staining was performed to determine the apoptotic effect of LicoA with or without siGRP78 in ELT3 cells. (**D**) MTT assay of cell viability. (**E**) ER stress response, as determined via ER-ID immunofluorescence staining. Scale bar = 50 μm. (**F**) Western blot analysis of the expression of GRP78, CHOP, c-caspase-3, c-caspase-9, c-PARP, p-NRF2, NRF2, and β-actin. * *p* < 0.05; ** *p* < 0.01 versus control (means ± SE; *n* = 3). # *p* < 0.05 versus LicoA treatment (mean ± SE; *n* = 3).

The excessive generation of ROS has been reported to cause ER stress-dependent apoptosis [37]. Therefore, we investigated whether LicoA induces ER stress in ELT3 cells. We observed that LicoA dramatically increases ER stress, as indicated by the increase in the intensity of the ER-ID green fluorescence (Figure 4A). Western blot assays further confirmed that the expression levels of the ER stress markers GRP78, IRE1α, ATF6, and CHOP were higher in LicoA-treated ELT3 cells than in untreated cells (Figure 4B). Glucose-Regulated Protein 78 (GRP78) is a key chaperone protein and participant in the unfolded protein response (UPR) in the endoplasmic reticulum (ER); it is also involved in apoptosis-related signaling pathways [38]. We observed that GRP78 knockdown (siGRP78) significantly decreased LicoA-induced apoptosis from 42.50% to 22.70% (Figure 4C) and restored the viability of cells (Figure 4D); it also decreased ER stress (Figure 4E) and the expression of ER stress-related proteins (GRP78/CHOP), apoptotic proteins (c-caspase-3, c-caspase-9 and c-PARP), p-NRF2, and NRF2 in ELT3 cells (Figure 4F). These findings suggest that LicoA induces the ER stress-mediated apoptosis of ELT3 cells via GRP78/p-NRF2 pathways.

### 3.5. Altered JNK/GRP78/p-NRF2 Expression Is Involved in LicoA-Induced ER Stress-Mediated ELT3 Cell Apoptosis

**Figure 5:**
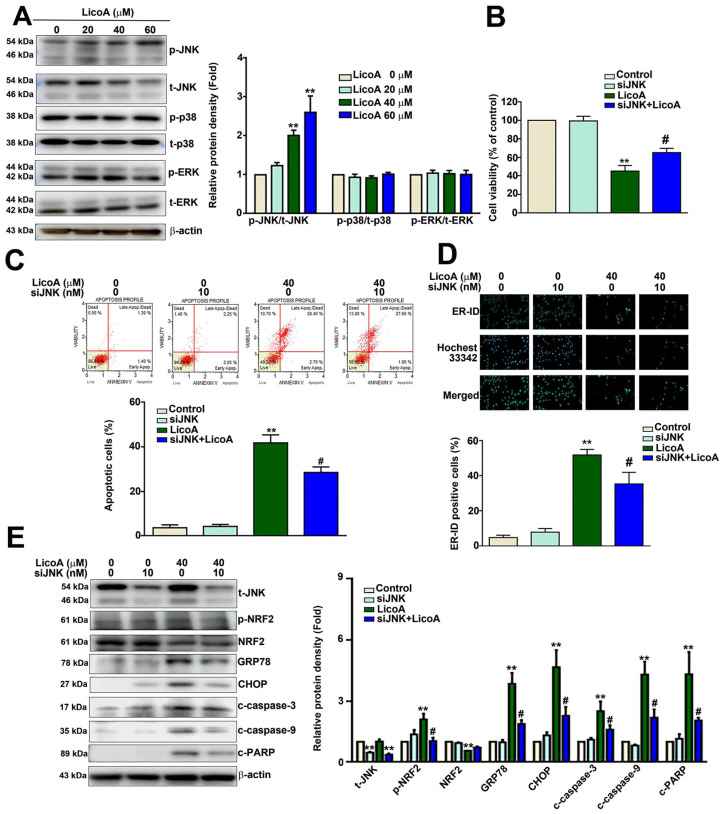
ER stress induction of apoptosis by LicoA via the JNK/NRF2 signaling pathway. (**A**) LicoA-induced activation of the MAPK (JNK, p38, and ERK) pathways in ELT3 cells, as determined via Western blotting. (**B**) Cell viability, as determined via MTT assay. (**C**) Flow cytometry of annexin-V/PI-stained ELT3 cells, which was performed to determine the apoptotic effect of LicoA with or without siJNK. (**D**) ER stress, as determined via ER-ID immunofluorescence staining. Scale bar = 50 μm. (**E**) The expression of t-JNK, p-NRF2, NRF2, GRP78, CHOP, c-caspase-3, c-caspase-9, c-PARP, and β-actin, as determined via Western blotting. ** *p* < 0.01 versus control; # *p* < 0.05 versus LicoA treatment (means ± SE; *n* = 3).

The MAPK signaling network is involved in cell proliferation, apoptosis, and ER stress [39]. To determine which MAPK signaling proteins are involved in the LicoA-induced inhibition of ELT3 cell growth, we investigated the activity of the MAPK signaling pathway in LicoA-exposed leiomyoma ELT3 cells using Western blotting. We found that treatment with LicoA upregulated the expression of p-JNK without altering the expression of p-p38 and p-ERK in ELT3 cells (Figure 5A). JNK knockdown using siJNK in ELT3 cells significantly reversed the LicoA-induced decrease in cell viability (Figure 5B), decreased the apoptotic effect (Figure 5C), and decreased ER stress (Figure 5D). In addition, siJNK decreased the expression of JNK, p-NRF2, ER stress-related proteins (GRP78/CHOP), and apoptotic proteins (c-caspase-3, c-caspase-9, and c-PARP) in LicoA-treated ELT3 cells (Figure 5E). These findings indicate that the activation of the JNK signaling may be involved in LicoA-induced ER stress-dependent apoptosis in ELT3 cells.

### 3.6. Uterine Leiomyoma Growth Inhibition by LicoA In Vivo

**Figure 6:**
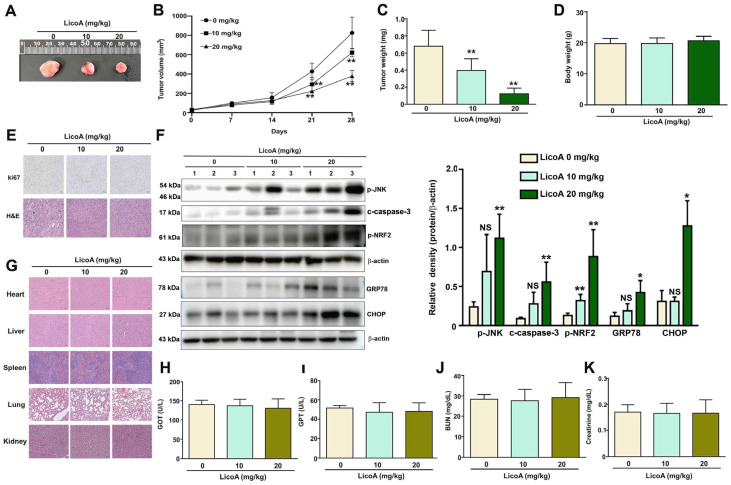
Anti-tumor effect of LicoA on UL growth in immunodeficient mice. Animal assays of UL growth using ELT3 cells subcutaneously injected into the immunodeficient BALB/c nude mice. The mice were then administered LicoA (0, 10, and 20 mg/kg) via oral gavage for 28 days. (**A**) Representation of the tumor tissues of the mice in each group. (**B**) The tumor volume was calculated for the three groups of mice. (**C**) The tumor weight and (**D**) body weights of the mice were measured. (**E**) H&E (hematoxylin and eosin) staining was performed, and the expression of Ki67 was detected via the IHC staining of tumor tissues. Scale bar = 50 μm. (**F**) The expression of p-JNK, p-NRF2, ER stress-related proteins (GRP78/CHOP), and apoptotic proteins (c-caspase 3) in the ELT3 tumor tissue of mice, as determined via Western blotting. (**G**) The heart, kidneys, liver, and spleen of the mice were fixed in neutral-buffered formalin and stained with H&E to evaluate the toxicity of LicoA (200× magnification). Blood analysis of (**H**) GOT, (**I**) GPT, (**J**) BUN, and (**K**) creatinine in all mice. * *p* < 0.05; ** *p* < 0.01 vs. control group (means ± SE; *n* = 5).

Five-week-old immunodeficient BALB/c nude mice were randomly divided into three groups (*n* = 5 per group): a control group, a LicoA 10 mg/kg group, and a LicoA 20 mg/kg group. The mice were subcutaneously injected with ELT3 cells (1 × 10^7^/100 μL) and then administered LicoA orally (0, 10, or 20 mg/kg) for 28 days. The growth of tumors in the mice was observed (Figure 6A). We found that LicoA significantly decreased tumor growth (Figure 6B) and tumor weight (Figure 6C), with no effect on body weight (Figure 6D). The HE staining and immunohistochemical staining of Ki67 revealed a notable reduction in tumor mass in the mice treated with LicoA (10 and 20 mg/kg) compared to the control group (Figure 6E). The LicoA-upregulated expression of p-JNK, p-NRF2, ER stress proteins (GRP78/CHOP), and apoptotic proteins (c-caspase-3) was observed in the ELT3 tumor tissue of the mice (Figure 6F). An examination of the heart, liver, spleen, lungs, and kidneys of the mice revealed no LicoA-induced toxicity and no injuries (Figure 6G). The blood analysis results show that LicoA did not influence the GOT (Figure 6H), GPT (Figure 6I), BUN (Figure 6J), or creatinine (Figure 6K) levels in mice. These results suggest that LicoA exerts anti-growth effects against uterine leiomyoma in vivo.

## 4. Discussion

**Figure 7:**
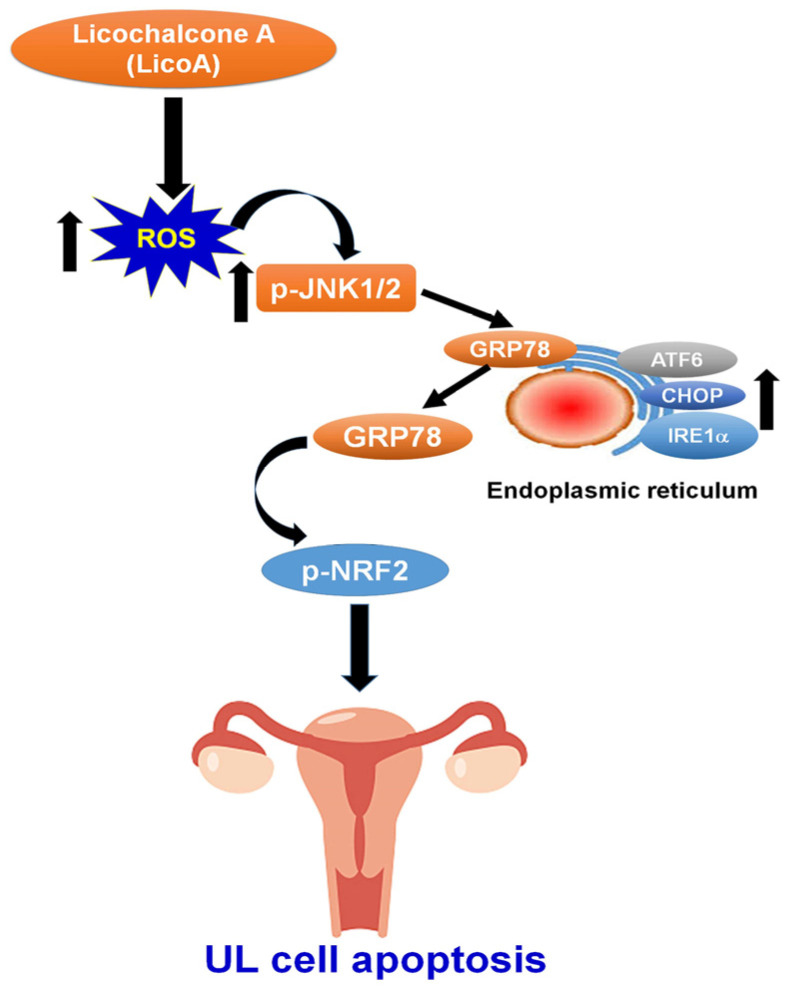
Schematic overview of the mechanism via which LicoA exerts an anti-tumor effect on UL cells. LicoA induces ROS-mediated UL cell apoptosis, resulting in the induction of JNK and the activation of the GRP78/p-NRF2 axis.

The ability of many naturally occurring plant compounds to prevent and treat tumors has been investigated in preclinical and clinical trials. This study explored the therapeutic potential and molecular mechanisms underlying the treatment of uterine leiomyoma with LicoA. The findings of this study show that (i) the LicoA treatment inhibited the growth of uterine leiomyoma cells and induced cell apoptosis; (ii) the LicoA treatment significantly increased intracellular ROS-mediated ER stress by increasing the expression of p-NRF2 and ER stress-related proteins (GRP78, IRE1α, ATF6, and CHOP); (iii) JNK activation was responsible for the LicoA-induced suppression of cell growth, ER stress induction, and changes in the expression of p-NRF2, GRP78, CHOP, and apoptotic proteins (c-caspase 3, c-caspase 9, and c-PARP); and (iv) LicoA possesses anti-tumor properties, further confirmed via an analysis of the growth of UL in immunodeficient mice in vivo. These results suggest that LicoA induces UL cell apoptosis via ROS-mediated JNK activation by activating the GRP78/p-NRF2 pathway in vitro and in vivo (Figure 7).

Previous studies have shown that LicoA exhibits anti-tumor activities in several types of cancer. LicoA dramatically inhibited cell growth and induced apoptosis via the upregulation of GRP78 expression and ER stress in endometrial cancer [34]. LicoA was also found to suppress cell survival, cause cell cycle arrest, and inhibit the motility of invasive cells by modulating the FAK/Src-mediated expression of Sp1 in renal cell carcinoma (RCC) [34]. In osteosarcoma cells, LicoA exhibited anti-proliferation effects and induced apoptosis through the p38MAPK-mediated mitochondrial apoptosis pathway in vitro and in vivo [40]. LicoA was shown to exert anti-invasive properties against human glioma by targeting the MEK/ERK and ADAM9 pathways [41]. In hepatocellular carcinoma cells, LicoA was shown to inhibit the activity, migration, and invasion of urokinase-type plasminogen activator (uPA) by targeting the binding ability of NF-κB to the uPA promoter in the MKK4/JNK-dependent pathway [42]. Furthermore, co-treatment with LicoA and Sor resulted in significant synergistic anti-metastatic effects on human hepatocellular carcinoma via the inactivation of the MKK4/JNK pathway and the downregulation of uPA [43]. In the present study, LicoA treatment inhibited cell growth at lower doses in ELT3 cells than in UtSMCs. These results indicate that LicoA exerts highly selective and specific anti-tumor activity against UL cells. In in vitro and in vivo studies, LicoA induced UL cell apoptosis, mainly via ROS-mediated ER stress and the JNK-dependent pathway.

ROS play an important role in maintaining homeostasis under conditions of cellular oxidative stress [44]. Therefore, targeting ROS production could enhance the treatment of tumors. The generation of ROS by protodioscin has been suggested as a strategy for the treatment of cervical cancer through ROS-mediated ER stress and apoptosis [37]. ROS have been shown to mediate the anti-UF activity of nerolidol via DNA damage and G1-phase cell cycle arrest by downregulating the ATM/AKT pathway [45]. LicoA decreased cell viability and induced G2/M-phase cell cycle arrest in ovarian cancer cells. LicoA mainly exerted anticancer activity via ROS-mediated mitochondrial dysfunction and apoptosis [46]. LicoA activated the ULK1/Atg13 complex to induce autophagy and apoptosis via the generation of ROS in human hepatocellular carcinoma cells [47]. The activation of ROS by Manzamine A may be a promising therapeutic approach to suppressing UL cell growth and matrix deposition by inhibiting sterol o-acyltransferases [48]. Gyejibongnyeong-hwan has been reported to significantly ameliorate UL by increasing the concentration of mitochondrial ROS and apoptosis level [49]. The phytochemical deoxycortin exhibits inhibitory effects on UL growth by inducing G2/M-phase cell cycle arrest, ROS-dependent caspase-3-mediated mitochondrial intrinsic apoptosis, and the downregulation of oncogenic lncRNA [50]. In the present study, we observed, for the first time, that LicoA inhibits the growth of UL cells by inducing apoptosis through ROS-mediated ER stress.

ER stress is associated with the death of tumor cells. The induction of tumor cell apoptosis through ER stress can be used to treat tumors. Liquiritin exerts immunogenic cell death (ICD) effects on human cervical cancer by inducing HMGB1 release, ATP secretion, and calreticulin exposure through ER stress [51]. Protodioscin inhibits the proliferation of cells and causes mitochondrial dysfunction and apoptotic effects in cervical cancer cells via ROS-upregulated ER stress. Protodioscin has been shown to significantly upregulate the expression of the ER stress-responsive proteins GRP78, ATF4, p-eIF-2α, and CHOP. Protodioscin also promoted the binding of ATF4 to the CHOP promoter, thus contributing to tumor apoptosis [37]. Norcantharidin (NCTD) exerts anti-tumor effects such as growth suppression, mitochondrial depolarization, and apoptosis induction in renal cancer cells through ER stress; this is accompanied by an increase in the expression of GRP78, p-elF2α, ATF4, and the CHOP protein in vivo and in vitro [52]. LicoA induces G2/M-phase cell cycle transition by decreasing the expression of MDM2, cyclin B1, cdc2, and cdc25c proteins, and it causes apoptosis by upregulating the activation of proapoptotic caspase-3 and PARP cleavage via ER stress in human lung cancer [53]. LicoA exerts anticancer effects by inducing the apoptosis of endometrial cancer cells (EMCs) via the GRP78-mediated ER stress pathway [34]. Here, we present the first evidence that LicoA is a critical moderator of ER stress in UL, indicating its therapeutic potential.

Many studies have reported that MAPK pathways are potential therapeutic targets for tumor treatment. The curcumin analog HO-3867 has been shown to activate caspase 3, caspase 8, caspase 9, and PARP via the JNK pathway, resulting in the apoptosis of human oral cancer cells [54]. Fisetin has been shown to suppress cell proliferation through G2/M-phase cell cycle arrest, the downregulation of cyclin D1, and the upregulation of p21/p27 in renal cell carcinoma (RCC). Coronarin D caused significant mitochondrial dysfunction, while the targeting of the JNK pathway by coronarin D contributed to the inhibition of HCC progression [55]. Curcumol has been reported to attenuate the development of uterine leiomyoma by inhibiting cell proliferation and migration and by inducing apoptosis through the p38MAPK and NF-κB pathways [56]. This study is the first to report that LicoA activates the JNK pathway by modulating the GRP78/p-NRF2 pathway-mediated apoptosis of uterine leiomyoma cells. However, further studies are needed to determine whether the effects of LicoA on p-NRF2 impact the transcriptional activity of CHOP or apoptotic gene expression in UL cells.

Several studies have explored the clinical applications and effects of LicoA, particularly in the fields of oncology and dermatology. Research on breast tissue samples from high-risk ethnic groups has suggested that LicoA could be used as a preventive agent against breast cancer [57]. Clinical trials have also investigated the anti-inflammatory properties of LicoA in sunscreen formulations, particularly for acne-prone patients. LicoA effectively reduced inflammation and reduced the severity of acne within four weeks, highlighting its potential use as an adjunctive therapy for skin conditions aggravated by UV exposure [58,59,60]. These findings underscore the potential use of LicoA in diverse clinical applications, ranging from the enhancement of skin health to cancer prevention. However, further studies are necessary to validate its efficacy and therapeutic feasibility, particularly in patients with UL.

## 5. Conclusions

This study demonstrates that LicoA effectively induces apoptosis in uterine leiomyoma cells by activating the JNK signaling pathway through the modulation of the GRP78/p-NRF2 axis. These findings highlight the potential application of LicoA as a novel and promising anticancer agent for the treatment of uterine leiomyoma, paving the way for future research and advances in uterine leiomyoma.

## Data Availability

The data that support the findings of this study are available from the corresponding author upon reasonable request.
